# Comparison of Hematological and Biochemical Results Derived from Arterial and Venous Blood Samples in Post-Anesthetic Dogs

**DOI:** 10.3390/ani10112069

**Published:** 2020-11-09

**Authors:** Song Mi Lee, Byung-Jae Kang, Sungin Lee, Wan Hee Kim

**Affiliations:** Department of Veterinary Clinical Sciences, College of Veterinary Medicine and Research Institute for Veterinary Science, Seoul National University, 1 Gwanak-ro, Gwanak-gu, Seoul 08826, Korea; songmi77@snu.ac.kr (S.M.L.); bjkang81@snu.ac.kr (B.-J.K.)

**Keywords:** arterial, biochemical, dog, hematological, interchangeability, venous

## Abstract

**Simple Summary:**

In contrast to humans, general anesthesia is required for both surgical procedures and diagnostic imaging like magnetic resonance imaging (MRI) and computed tomography (CT) in small animals. Because the post-anesthetic period is a particularly high risk time for death, serial monitoring of biochemical and hematological parameters could be required. Blood samples could be collected from an indwelling arterial catheter to avoid stress caused by venipuncture, but studies using arterial blood for hematological and biochemical testing have been limited. The aim of this study is to compare hematological and biochemical results derived from venous and arterial blood samples, and to evaluate their clinical interchangeability in post-anesthetic dogs. We found statistically significant but clinically irrelevant differences in hemoglobin, glucose, creatinine, and calcium levels, and results from venous and arterial blood samples are not clinically interchangeable for gamma-glutamyl transpeptidase and potassium levels.

**Abstract:**

Collecting blood from an indwelling arterial catheter may reduce stress from repeated venipuncture in patients requiring serial monitoring, but the use of arterial blood for hematological and biochemical testing remains understudied. Here, we compared hematological and biochemical results of arterial and venous blood and evaluated their clinical interchangeability. Blood samples from dogs who had recovered from anesthesia, collected by both arterial catheterization and venipuncture, were analyzed. To assess clinical acceptance between paired samples, the limit of agreement between the values derived from the arterial and venous blood samples was compared with the allowable total error (TEa) recommended for each parameter. We found no significant differences between the arterial and venous sample results for red/white blood cell and platelet counts and hematocrit, blood urea nitrogen, phosphate, total protein, albumin, alanine aminotransferase, aspartate aminotransferase, alkaline phosphatase, gamma-glutamyl transpeptidase, total bilirubin, sodium, potassium, and chloride levels, whereas hemoglobin, glucose, creatinine, and calcium levels differed significantly (*p* < 0.05). Moreover, only gamma-glutamyl transpeptidase and potassium exceeded the recommended TEa. Hematological and biochemical results derived from venous and arterial blood samples are clinically interchangeable in post-anesthetic dogs, with the exception of gamma-glutamyl transpeptidase and potassium; thus, these values should be used with caution.

## 1. Introduction

Laboratory test results may be affected by several factors other than disease, such as experimental procedures, sampling technique, body posture, circadian variation, diet, stress, improper handling and storage, and analytical imprecision and inaccuracy [1,2]. Previous studies have repeatedly questioned the effect of the sampling site on the measured values of several laboratory analyses in various species [2,3,4]. Blood samples are commonly collected from easy-to-access veins (i.e., the jugular and cephalic veins), and reference ranges for various analyses have been established based on the results of venous blood samples [2,5,6].

In contrast to human medicine, general anesthesia is required not only for surgery, but also for diagnostic imaging like magnetic resonance imaging (MRI) and computed tomography (CT) in small animals [7]. During general anesthesia, peri-anesthetic complications such as hypoventilation, hypotension, and arrhythmia can occur depending on the patient’s underlying disease, age, breed, and type of procedure [8,9]. Various parameters have been evaluated to identify such complications during anesthesia; in particular, arterial catheterization (A-line) is often performed on patients for the accurate monitoring of blood pressure, respiratory function, and acid/base status [8,10]. Since peri-anesthetic complications can lead to death [11], and the post-anesthetic period is of particularly high risk, intensive care is recommended during this time [12].

In critically ill patients, serial monitoring of blood components could be required. Repeated venipuncture can be more stressful for patients who are already stressed, and more time and labor could be required for patients who strongly resist [13]. To minimize these adverse effects, blood samples are often collected from indwelling catheters [14]. An arterial catheter is often sustained in critically ill patients after surgery or anesthesia to evaluate blood pressure or respiratory function. Because repetitive punctures are needed if venous blood samples are used for tests, blood collection through the A-line can benefit both the patients and the intensive care unit staff [13].

As previously explained, however, most reference ranges of blood tests in dogs are based on the results of venous blood samples; hence, it is necessary to validate whether results derived from arterial blood samples are reliable. The purpose of this study was to explore whether arterial and venous blood samples can be used interchangeably to measure commonly analyzed canine blood components.

## 2. Materials and Methods

### 2.1. Animals

Fifty-four client-owned dogs admitted to the Veterinary Medicine Teaching Hospital at Seoul National University (SNU) from December 2019 to April 2020 for a surgical procedure or diagnostic imaging were enrolled in the study. A sample size of 50 was recommended as the minimum sample size for a method comparison study [15]. Informed consent was obtained from the dogs’ owners and all procedures were approved by the SNU Institutional Animal Care and Use Committees (SNU-191025-14).

The study was conducted on dogs who had recovered from anesthesia for surgery or imaging. The indications of surgery or diagnostic imaging varied. If the anesthesia was repeated at different times, the samples were considered to differ even if they were obtained from the same dog.

### 2.2. Anesthesia

The dogs were premedicated with intravenous (IV) injection of either acepromazine (10–20 μg/kg, Sedaject, Samu Median) and medetomidine (2 μg/kg, Domitor, Orion), or midazolam (0.1–0.2 mg/kg, Midazolam, Bukwang Pharm) according to the dog’s American Society of Anesthesiologists grade. Induction of anesthesia was achieved with propofol (4–6 mg/kg IV, Profol, Baxter) or alfaxalone (2 mg/kg IV, Alfaxan, Jurox), and all dogs were maintained on isoflurane (Ifran, Hana Pharm). During anesthesia, glycopyrrolate (5 μg/kg IV, Mobinul, Myungmoon) was administered as needed in case of bradycardia, and ephedrine (0.1 mg/kg IV, Ephedrine HCl, Jeil Pharmaceutical) was administered to dogs whose blood pressure was low. Analgesia was provided with remifentanil (0.1 μg/kg/minute, Remiva, Hanapharm, continuous rate infusion, IV) or hydromorphone (0.05 μg/kg IV, Dilid, Hanapharm).

### 2.3. Blood Collection

After the injection of premedication, a 22–24 G catheter (Bio-Safety I.V catheter, Sewoon Medical) was placed in the dorsal pedal artery. The dogs recovered from anesthesia after a scheduled surgery or diagnostic procedure. Blood samples were collected after full recovery from the anesthesia. Because perfusion failure can be induced under conditions of hemodynamic instability such as shock [16,17], mean arterial pressure (MAP), temperature (T), pulse rate (P), respiratory rate (R), and capillary refill time (CRT), i.e., the traditional physical examination parameters for the evaluation of hemodynamic instability [18], were measured at the time of blood sampling to eliminate this effect on the findings of the study. The CRT was evaluated by one person to avoid inter-observer variability.

The venous blood sample was collected from the external jugular vein using a 23 G, 3 mL syringe (KOVAX-SYRINGE, Korea Vaccine), and the arterial blood sample was collected through the A-line after discharging 4 mL (>3 times of dead space volume) [19,20]. The sequence of the site for blood collection was determined by the toss of a coin. All blood samples were collected by the same person.

At least 1.5 mL of blood was drawn with a 3 mL syringe (KOVAX-SYRINGE, Korea Vaccine); 0.5 mL of blood was transferred to an ethylenediamine tetraacetic acid (EDTA) tube (1.3 mL K3 EDTA, Sarstedt), and 1 mL of blood was transferred to a heparin tube (Fuji heparin tube, Fujufilm). The sampling interval between the arterial and venous blood collections was less than 5 min, and samples collected with longer intervals were excluded.

EDTA-stabilized blood was used for the complete blood count, which was maintained at room temperature and analyzed within 4 h of sampling. Heparinized blood was centrifuged at 3000× *g* for 5 min, and plasma was separated within 1 h of sampling. Biochemical analysis was performed within a day after sampling. Samples with hemolysis, lipemia, or icteric discoloration were excluded.

### 2.4. Analytical Procedures

Hematological analysis included red blood cell count (RBC), hematocrit, hemoglobin, white blood cell count (WBC), and platelet count, determined using a hematology analyzer (ProCyte Dx, IDEXX).

Biochemical analysis included glucose, creatinine, blood urea nitrogen (BUN), phosphate, calcium, total protein (TP), albumin, alanine aminotransferase (ALT), aspartate aminotransferase (AST), alkaline phosphatase (ALP), gamma-glutamyl transpeptidase (γ-GT), total bilirubin, sodium (Na), potassium (K), and chloride (Cl) levels, which were determined using a chemistry analyzer (Catalyst One, IDEXX).

The analyzers were maintained by regular quality control procedures and managed according to the manufacturers’ instructions.

### 2.5. Statistical Analysis

Data were analyzed with statistical software (GraphPad Prism 8.4.2, GraphPad Software) and are presented as means and standard deviations or range. The differences between paired results from venous and arterial blood were analyzed using a paired *t*-test (confirmed normality) or Wilcoxon signed rank test (failed normality). The level of significance was set at *p* < 0.05.

Bland–Altman analysis was used to evaluate the agreement between the blood collection sites. To assess clinical acceptance between paired samples, the allowable total error (TEa) for each parameter, as defined by the American Society for Veterinary Clinical Pathology (ASVCP) guidelines, was compared with the limit of agreement (LOA) derived from the study.

## 3. Results

### 3.1. Animals and Samples

Fifty-nine paired samples from 54 dogs were collected. Of those, 51 paired samples from 47 dogs were included in the study, excluding samples with visible hemolysis, lipemia, or icteric discoloration. Two paired samples were excluded due to arterial hemolysis, two due to venous hemolysis, and two due to both arterial and venous hemolysis. One paired sample each was excluded due to lipemia and bilirubinemia.

The mean age and weight of the 47 dogs was 7.34 years (range 0.4–14 years) and 12.17 kg (range 2.5–47.9 kg), respectively. Twenty-five dogs were castrated males, 4 were intact males, 14 were spayed females, and 4 were intact females. Of the 51 paired samples, 32 were obtained after surgery and 19 were obtained after diagnostic imaging.

The MAP, T, P, R, and CRT of the dogs, measured at the time of blood collection, are shown in Table 1 and were within the normal range in all dogs.

In the case of total bilirubin, as a concentration lower than 0.1 mg/dL cannot be measured with the analyzer, the difference in the results between the venous and arterial blood samples could only be compared in 14 paired samples. Bland–Altman analysis was not conducted because the sample size did not suffice. Results lower than 10 U/L for ALT and higher than 2000 U/L for ALP cannot be measured with the analyzer; thus, 50 paired samples were included, excluding one paired sample each.

### 3.2. Hematological Parameters

There was no significant difference in RBC, hematocrit, WBC, and platelet count between values derived from arterial and venous blood samples, whereas the hemoglobin level was significantly higher in the venous than in the arterial sample (*p* = 0.008) (Table 2).

**Figure 1:**
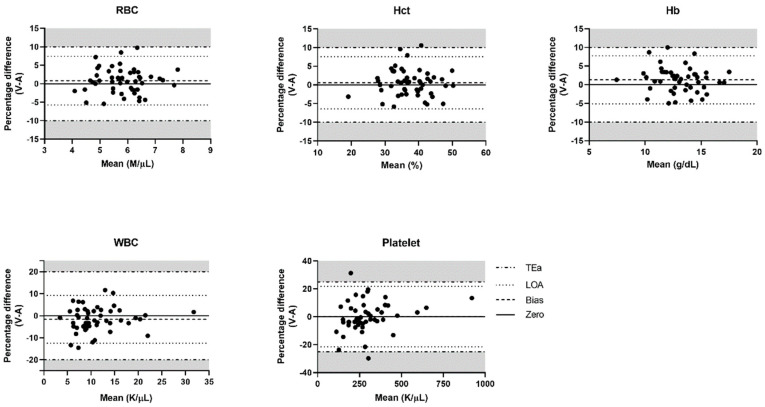
Bland–Altman plots of hematological parameters comparing arterial and venous blood samples. The percentage difference of venous and arterial results is plotted against the mean of the venous and arterial results. Gray shading indicates the area exceeding the allowable total error (TEa). If the upper and lower lines of the LOA are not within the gray shading, it indicates clinical interchangeability between the venous and arterial results. RBC, red blood cell; Hct, hematocrit; Hb, hemoglobin; WBC, white blood cell; LOA, limit of agreement.

Bland–Altman plots of the RBC, hematocrit, hemoglobin, WBC, and platelet results were constructed from the venous–arterial pairs (Figure 1). Results for bias, LOA, and TEa for each hematological parameter defined by the ASVCP guidelines are summarized in Table 3. The comparison of the LOA for each parameter with its TEa showed that the LOAs of all hematological parameters were within the proposed limits (Figure 1).

### 3.3. Biochemical Parameters

Differences between arterial and venous sample results were only noted for glucose, creatinine, and calcium (Table 2).

**Figure 2:**
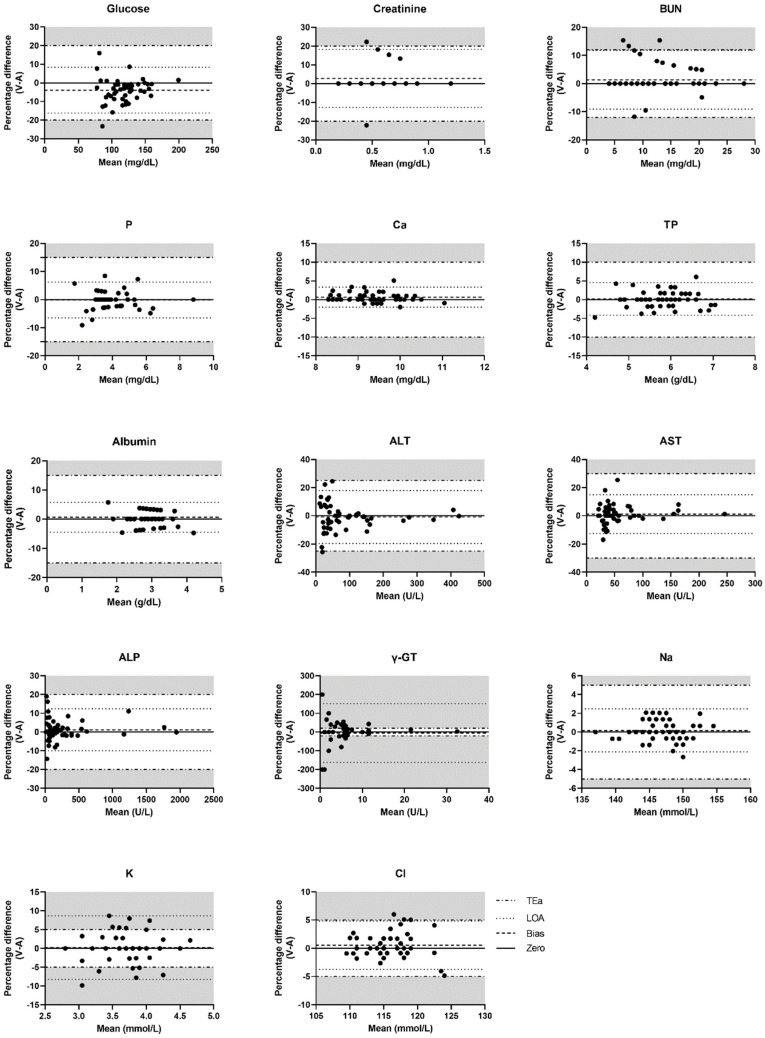
Bland–Altman plots of biochemical parameters comparing arterial and venous blood samples. The percentage difference of venous and arterial results is plotted against the mean of the venous and arterial results. Gray shading indicates the area exceeding the allowable total error (TEa). If the upper and lower lines of the LOA are not within the gray shading, it indicates clinical interchangeability between the venous and arterial results. BUN, blood urea nitrogen; P, phosphate; Ca, calcium; TP, total protein; ALT, alanine aminotransferase; AST, aspartate aminotransferase; ALP, alkaline phosphatase; γ-GT, gamma-glutamyl transpeptidase; Na, sodium; K, potassium; Cl, chloride; LOA, limit of agreement.

Bland–Altman plots of the glucose, creatinine, BUN, phosphate, calcium, TP, albumin, ALT, AST, ALP, γ-GT, Na, K, and Cl results were constructed from the venous–arterial pairs (Figure 2). The results for bias, LOA, and TEa for each biochemical parameter as defined by the ASVCP guidelines are summarized in Table 3. The comparison of the LOA for each parameter with its TEa showed that the LOAs of γ-GT and K were not within the proposed limits, while the LOAs of the other biochemical parameters were within their TEa (Figure 2).

## 4. Discussion

In this study, hematological and some biochemical parameters obtained from the A-line of the dorsal pedal artery and venipuncture of the jugular vein were compared. The only significant differences between the arterial and venous blood sample results were in hemoglobin, glucose, creatinine, and calcium levels. Agreement was found for most analytes investigated in this study, except for K and γ-GT.

Studies exploring the effects of sampling site on test results have been conducted on various animal species, sampling sites, and parameters. Orlowski et al. [21] reported identical hemoglobin results derived from arterial and venous blood. Son et al. [22] confirmed that there were no significant differences in hematocrit results derived from arterial and venous blood collected from anesthetized dogs and healthy humans. However, results can be influenced by the selected and compared sampling sites [23]. Palsgaard-Van Lue et al. [13], who used the same set of blood sampling sites as ours to compare arteriovenous hematology in dogs, reported that WBC, hemoglobin, RBC, hematocrit, and platelet count were significantly lower in arterial than in venous blood. Their results regarding hemoglobin agreed with ours, but those pertaining to the other parameters differed, possibly because they collected arterial blood after the induction of anesthesia with propofol, which is known to promote a reduction in RBC, hemoglobin, hematocrit, and WBC in dogs [24].

Orlowski et al. [21] compared the values of Na, K, Cl, BUN, creatinine, calcium, phosphorus, TP, albumin, glucose, total bilirubin, ALP, and AST of blood collected from an artery, vein, and bone marrow, and found that only the glucose level was marginally significantly higher in arterial blood, which is in line with our glucose-related results and could be explained by the well-recognized physiological fact that some glucose diffuses from the plasma into the interstitial fluid during circulation through the capillary system, and thus its levels are higher in arterial blood [25,26,27].

Slot [28] reported a significant arteriovenous difference in endogenous creatinine, with a higher concentration in venous than in arterial blood in humans. Slot [28] explained that this is because creatinine is produced by muscles, and subsequently transported by venous blood. Similarly, a previous study [2] that compared the creatinine level between the jugular and cephalic veins in dogs found a significantly higher creatinine level in the jugular vein, which collects blood from the muscles, than in the cephalic vein.

However, significant differences in hemoglobin, glucose, creatinine, and calcium are not necessarily clinically unacceptable. Likewise, the lack of significant differences in the other parameters does not signify that the values are interchangeable. The statistical method suggested by Altman and Bland in 1983 [29] is recommended to determine whether two tests are clinically interchangeable. This technique uses the set of differences between two tests and their standard deviations (SDs) to calculate an agreement interval (known as LOA). The calculated width of the LOA is then compared with an interval considered acceptable in clinical practice. If the LOA falls entirely within the clinically acceptable interval, then the two tests may be used interchangeably [30,31]. Clinically acceptable intervals based on differences that lead to different judgements for treatment are defined as TEa and vary depending on species, the concentration of the samples, and the parameters [32,33]. Some acceptable clinical limits of laboratory analytes are used in human medicine (e.g., the Clinical Laboratory Improvement Amendments criteria, European recommendations) [34]. However, the only available criterion in veterinary medicine is the ASVCP guideline [32,33].

Comparing the TEa of each parameter supplied by the ASVCP with the LOA derived from this study, the LOAs of K and γ-GT did not entirely fall within their TEas. In cats, statistically significant but clinically irrelevant differences in K, TP, and albumin concentration were observed between blood samples obtained by direct venipuncture and a venous access port [35]. Moreover, another study on dogs [19] reported a statistically significant but clinically irrelevant difference in K between blood samples collected by a push–pull technique from an indwelling catheter and direct venipuncture. The two previous studies suggested the possibility of hemolysis during blood collection as the cause of the difference. In vitro hemolysis is known to increase the K concentration in the serum or plasma [36]. Hemolysis was visually checked and excluded from the present study. However, as it is challenging to completely visually exclude small amounts of hemolysis [37], it may be assumed that the small amount of hemolysis that occurred during venipuncture or arterial blood sampling via the catheter produced no significant difference but clinically unacceptable variation.

Another possible reason for such variation is that cells, which contain 98% of the K in the body, may have affected blood K concentration by altering K in/out flow according to the body condition. If the blood pH decreases, it affects various channels on the cell membrane, resulting in H^+^ inflow and K^+^ outflow. In contrast, if the blood pH increases, it stimulates H^+^ outflow and K^+^ inflow [38]. Meanwhile, in the RBC membrane, there is KCl co-transport that can induce K inflow into the cell [39], which is primarily controlled by PO_2_ and secondarily by pH [40,41]. Because K inflow increases when PO_2_ is high and when pH decreases to a certain level, the concentration of K is affected by blood PO_2_ and pH [41]. However, as the arterial and venous blood pH and PO_2_ were not measured at the time of the blood sampling in this study, we cannot explain the change in K plasma concentration in relation to such factors. Further research is needed to explore the effect of PO_2_ and pH on the difference between venous and arterial K levels.

In the case of γ-GT, no explanation could be found for this variation. γ-GT is evaluated in conjunction with hepatic parameters, especially ALP, as γ-GT alone appears to be of limited value for the diagnosis of canine liver disease [42]. Considering that hepatic parameters such as AST, ALT, and ALP acquired clinical acceptance between arterial and venous blood in the present study, we believe that the value of arterial γ-GT could be judged based on other results of hepatic parameters.

This study has a couple of limitations. First, some bias between sampling sites may be physiologically expected as also found in previous studies [2,4,13]. Furthermore, in certain states such as shock when the circulation centralizes to vital organs, laboratory results obtained from peripheral or central circulation cannot be undoubtedly considered equivalent [43]. Likewise, as dogs were in a stable hemodynamic state in our study, it remains unclear whether our results can be directly extrapolated to different hemodynamic states. However, we believe that this study could be the basis for future studies in patients who are hemodynamically unstable, such as critically ill patients. Second, as this study was conducted on patients, we could not use the same anesthetic protocol for all dogs. However, it can be considered that this study instead reflected the actual clinical situation, in which the anesthesia protocol should be set differently for each patient, considering various factors.

## 5. Conclusions

For the hematological and some biochemical parameters (except for γ-GT and K) evaluated in this study, samples collected from venous and arterial blood were found to be clinically interchangeable in post-anesthetic dogs. γ-GT can be assessed based on arterial blood samples, given that its value could be judged based on other liver enzymes. However, in dogs who need strict monitoring of their K level, precautions are necessary when using the arterial value interchangeably with the venous value for serial monitoring. The clinical implication of our results is that arterial blood samples can be used to measure most hematological and biochemical parameters, which could help avoid unnecessary restrictions or pain, especially in patients who excessively resist, are excited, and eventually show dyspnea to venipuncture. Furthermore, it could be a significant improvement for animal welfare. Veterinary staff could also benefit from this procedure as they could collect blood samples through the A-line by themselves, which could reduce the time and labor required for the venipuncture of aggressive dogs.

## Figures and Tables

**Table 1:** Physical examination measured at the time of blood sampling.

	Value ^a^
MAP (mmHg)	100.8 ± 14.4
Temperature (°C)	38.37 ± 0.59
Pulse rate (/minutes)	133.4 ± 28.6
Respiratory rate (/minutes)	25.57 ± 3.52
CRT (s)	<2

MAP, mean arterial pressure; CRT, capillary refill time. ^a^ Values of MAP, temperature, pulse rate, and respiratory rate are presented as mean ± standard deviation. All CRT values are less than 2 s.

**Table 2:** Comparison of hematological and biochemical results collected from arterial and venous blood.

Variables	Venous ^a^	Arterial ^a^	*p*-Value	Reference Range
RBC (M/μL)	5.86 ± 0.83	5.81 ± 0.83	0.074	5.65–8.87
Hematocrit (%)	37.68 ± 6.30	37.47 ± 6.33	0.274	37.3–61.7
Hemoglobin (g/dL)	13.15 ± 1.97	12.99 ± 2.00	0.008 ^c^	13.1–20.5
WBC (K/μL)	11.19 ± 5.24	11.33 ± 5.22	0.105	5.05–16.76
Platelet (K/μL)	298.4 ± 150.4	294.3 ± 134.8	0.675 ^b^	148–484
Glucose (mg/dL)	116.5 ± 25.1	120.9 ± 24.6	<0.000 ^c^	74–143
Creatinine (mg/dL)	0.62 ± 0.20	0.60 ± 0.21	0.022 ^b, c^	0.5–1.8
BUN (mg/dL)	12.74 ± 5.24	12.58 ± 5.22	0.096 ^b^	7–27
Phosphate (mg/dL)	4.01 ± 1.21	4.01 ± 1.22	0.637 ^b^	2.5–6.8
Calcium (mg/dL)	9.36 ± 0.63	9.30 ± 0.65	0.001 ^b, c^	7.9–12
TP (g/dL)	5.85 ± 0.61	5.84 ± 0.61	0.513	5.2–8.2
Albumin (g/dL)	2.90 ± 0.44	2.88 ± 0.45	0.146	2.3–4.0
ALT (U/L)	91.16 ± 97.43	92.16 ± 97.82	0.102 ^b^	10–125
AST (U/L)	58.54 ± 44.98	57.54 ± 43.79	0.111 ^b^	0–50
ALP (U/L)	281.7 ± 418.0	277.1 ± 408.5	0.389 ^b^	23–212
γ-GT (U/L)	4.66 ± 5.98	4.34 ± 5.53	0.146	0–11
Total bilirubin (mg/dL)	0.16 ± 0.08	0.19 ± 0.06	0.189	0–0.9
Na (mmol/L)	146.9 ± 3.4	146.7 ± 3.5	0.291	144–160
K (mmol/L)	3.71 ± 0.39	3.70 ± 0.37	0.659	3.5–5.8
Cl (mmol/L)	116.2 ± 3.5	115.5 ± 3.6	0.086	109–122

RBC, red blood cell; WBC, white blood cell; BUN, blood urea nitrogen; TP, total protein; ALT, alanine aminotransferase; AST, aspartate aminotransferase; ALP, alkaline phosphatase; γ-GT, gamma-glutamyl transpeptidase; Na, sodium; K, potassium; Cl, chloride. ^a^ Values are presented as mean ± standard deviation. ^b^ Analytes that failed the normality checks were analyzed using the Wilcoxon signed rank test. ^c^ Statistically significant differences were detected between arterial and venous blood samples (*p* < 0.05).

**Table 3:** Bias and limit of agreement (LOA) of Bland–Altman analysis and recommended allowable total error (TEa).

Variables	Bias (%)	LOA (%)		ASVCP TEa (%)
		Lower	Upper	
RBC	0.85	−5.74	7.440	10
Hematocrit	0.56	−6.41	7.54	10
Hemoglobin	1.31	−5.13	7.75	10
WBC	−1.59	−12.42	9.25	20
Platelet	0.14	−21.47	21.75	25
Glucose	−3.87	−16.17	8.43	20
Creatinine	2.75	−12.72	18.22	20
BUN	1.36	−9.09	11.80	12
Phosphate	−0.16	−6.47	6.24	15
Calcium	0.67	−2.01	3.34	10
TP	0.19	−4.17	4.55	10
Albumin	0.61	−4.50	5.71	15
ALT	−0.82	−19.55	17.90	25
AST	1.20	−12.59	14.99	30
ALP	1.22	−9.99	12.42	20
γ-GT	−4.99	−161.6	151.6	20
Na	0.18	−2.12	2.47	5
K	0.23	−8.23	8.70	5
Cl	0.55	−3.73	4.84	5

ASVCP, American Society for Veterinary Clinical Pathology; RBC, red blood cell; WBC, white blood cell; BUN, blood urea nitrogen; TP, total protein; ALT, alanine aminotransferase; AST, aspartate aminotransferase; ALP, alkaline phosphatase; γ-GT, gamma-glutamyl transpeptidase; Na, sodium; K, potassium; Cl, chloride.

## References

[1] Irjala K.M., Gronroos P.E. (1998). Preanalytical and analytical factors affecting laboratory results. Ann. Med..

[2] Jensen A.L., Wenck A., Koch J., Poulsen J.S. (1994). Comparison of results of haematological and clinical chemical analyses of blood samples obtained from the cephalic and external jugular veins in dogs. Res.Vet. Sci..

[3] Evron S., Tress V., Ezri T., Szmuk P., Landau O., Hendel D., Schechter P., Medalion B. (2007). The importance of blood sampling site for determination of hemoglobin and biochemistry values in major abdominal and orthopedic surgery. J. Clin. Anesth..

[4] Yang Z.W., Yang S.H., Chen L., Qu J., Zhu J., Tang Z. (2001). Comparison of blood counts in venous, fingertip and arterial blood and their measurement variation. Clin. Lab. Haematol..

[5] Kley S., Tschudi P., Busato A., Gaschen F. (2003). Establishing canine clinical chemistry reference values for the Hitachi^®^ 912 using the International Federation of Clinical Chemistry (IFCC) recommendations. Comp. Clin. Path..

[6] Lumsden J.H., Mullen K., McSherry B.J. (1979). Canine hematology and biochemistry reference values. Can. J. Comp. Med..

[7] Hildebrandt I.J., Su H., Weber W.A. (2008). Anesthesia and other considerations for in vivo imaging of small animals. ILAR J..

[8] Bednarski R., Grimm K., Harvey R., Lukasik V.M., Penn W.S., Sargent B., Spelts K. (2011). AAHA anesthesia guidelines for dogs and cats. J. Am. Anim. Hosp. Assoc..

[9] Gruenheid M., Aarnes T.K., McLoughlin M.A., Simpson E.M., Mathys D.A., Mollenkopf D.F., Wittum T.E. (2018). Risk of anesthesia-related complications in brachycephalic dogs. J. Am. Vet. Med. Assoc..

[10] Sasaki K., Shiga T., Gomez de Segura I.A. (2019). Advantages of a novel device for arterial catheter securement in anesthetized dogs: A pilot randomized clinical trial. Front. Vet. Sci..

[11] Hicks J.A., Kennedy M.J., Patterson E.E. (2013). Perianesthetic complications in dogs undergoing magnetic resonance imaging of the brain for suspected intracranial disease. J. Am. Vet. Med. Assoc..

[12] Brodbelt D.C., Blissitt K.J., Hammond R.A., Neath P.J., Young L.E., Pfeiffer D.U., Wood J.L. (2008). The risk of death: The confidential enquiry into perioperative small animal fatalities. Vet. Anaesth. Analg..

[13] Palsgaard-Van Lue A., Jensen A.L., Strom H., Kristensen A.T. (2007). Comparative analysis of haematological, haemostatic, and inflammatory parameters in canine venous and arterial blood samples. Vet. J..

[14] Elliott K.F., Fleeman L.M., Rand J.S. (2010). Using 20-gauge percutaneous peripheral catheters to reliably collect serial 4-mL blood samples from conscious dogs. Aust. Vet. J..

[15] Altman D.G. (1991). Some common problems in medical research. Practical Statistics for Medical Research.

[16] Bose E.L., Hravnak M., Pinsky M.R. (2015). The interface between monitoring and physiology at the bedside. Crit. Care Clin..

[17] Weil M.H. (2005). Defining hemodynamic instability. Functional Hemodynamic Monitoring.

[18] Sevransky J. (2009). Clinical assessment of hemodynamically unstable patients. Curr. Opin. Crit. Care.

[19] Barr C.A., Gianotti G., Graffeo C.E., Drobatz K.J., Silverstein D.C. (2017). Effect of blood collection by the push-pull technique from an indwelling catheter versus direct venipuncture on venous blood gas values before and after administration of alfaxalone or propofol in dogs. J. Am. Vet. Med. Assoc..

[20] May M.L., Nolen-Walston R.D., Utter M.E., Boston R.C. (2010). Comparison of hematologic and biochemical results on blood obtained by jugular venipuncture as compared with intravenous catheter in adult horses. J. Vet. Intern. Med..

[21] Orlowski J.P., Porembka D.T., Gallagher J.M., Van Lente F. (1989). The bone marrow as a source of laboratory studies. Ann. Emerg. Med..

[22] Son K.H., Lim C.H., Song E.J., Sun K., Son H.S., Lee S.H. (2010). Inter-species hemorheologic differences in arterial and venous blood. Clin. Hemorheol. Microcirc..

[23] O’Brien M., Murphy M.G., Lowe J.A. (1998). Hematology and clinical chemistry parameters in the cat (*Felis domesticus*). J. Nutr..

[24] Costa P.F., Nunes N., Belmonte E.A., Moro J.V., Lopes P.C.F. (2013). Hematologic changes in propofol-anesthetized dogs with or without tramadol administration. Arq. Bras. Med. Vet. Zootec..

[25] Bose J.P. (1935). Arterial versus venous blood sugar: Arterio-venous sugar difference as a criterion of the severity of diabetes. Ind. Med. Gaz..

[26] Cengiz E., Tamborlane W.V. (2009). A tale of two compartments: Interstitial versus blood glucose monitoring. Diabetes Technol. Ther..

[27] Somogyi M. (1949). Studies of arteriovenous differences in blood sugar; effect of intravenous insulin and simultaneous glucose feeding. J. Biol. Chem..

[28] Slot C. (1965). The significance of the systemic arteriovenous difference in creatinine clearance determinations. Scand. J. Clin. Lab. Invest..

[29] Altman D.G., Bland J.M. (1983). Measurement in medicine: The analysis of method comparison studies. Statistician.

[30] Bland J.M., Altman D.G. (1986). Statistical methods for assessing agreement between two methods of clinical measurement. Lancet.

[31] Bland J.M., Altman D.G. (1999). Measuring agreement in method comparison studies. Stat. Methods Med. Res..

[32] Harr K.E., Flatland B., Nabity M., Freeman K.P. (2013). ASVCP guidelines: Allowable total error guidelines for biochemistry. Vet. Clin. Pathol..

[33] Nabity M.B., Harr K.E., Camus M.S., Flatland B., Vap L.M. (2018). ASVCP guidelines: Allowable total error hematology. Vet. Clin. Pathol..

[34] Petersen P.H., Stockl D., Blaabjerg O., Pedersen B., Birkemose E., Thienpont L., Lassen J.F., Kjeldsen J. (1997). Graphical interpretation of analytical data from comparison of a field method with reference method by use of difference plots. Clin. Chem..

[35] Henry C.J., Russell L.E., Tyler J.W., Buss M.S., Seguin B., Cambridge A.J., Moore M.E. (2002). Comparison of hematologic and biochemical values for blood samples obtained via jugular venipuncture and via vascular access ports in cats. J. Am. Vet. Med. Assoc..

[36] Leard B.L., Alsaker R.D., Porter W.P., Sobel L.P. (1990). The effect of haemolysis on certain canine serum chemistry parameters. Lab. Anim..

[37] Hawkins R. (2002). Discrepancy between visual and spectrophotometric assessment of sample haemolysis. Ann. Clin. Biochem..

[38] Aronson P.S., Giebisch G. (2011). Effects of pH on potassium: New explanations for old observations. J. Am. Soc. Nephrol..

[39] Lauf P.K., Adragna N.C. (2000). K-Cl cotransport: Properties and molecular mechanism. Cell. Physiol. Biochem..

[40] Gibson J.S., Cossins A.R., Ellory J.C. (2000). Oxygen-sensitive membrane transporters in vertebrate red cells. J. Exp. Biol..

[41] Honess N.A., Gibson J.S., Cossins A.R. (1996). The effects of oxygenation upon the Cl-dependent K flux pathway in equine red cells. Pflugers Arch..

[42] Shull R.M., Hornbuckle W. (1979). Diagnostic use of serum gamma-glutamyltransferase in canine liver disease. Am. J. Vet. Res..

[43] Jousi M., Laukkanen-Nevala P., Nurmi J. (2019). Analysing blood from intraosseous access: A systematic review. Eur. J. Emerg. Med..

